# Fluctuations of antimitochondrial antibodies and anti-gp210 antibody in a patient with primary biliary cholangitis and Sjögren syndrome with subsequent autoimmune hemolytic anemia: A case report

**DOI:** 10.1097/MD.0000000000018856

**Published:** 2020-01-17

**Authors:** Dan-Tong Zhao, Yan-Min Liu, Ying Han, Hai-Ping Zhang, Yan Zhao, Hui-Ping Yan

**Affiliations:** a Clinical Research Center for Autoimmune Liver Disease & Clinical Laboratory Center; b Department of Liver Disease Immunology, Beijing You’an Hospital, Capital Medical University, Beijing, China.

**Keywords:** anti-gp210 antibody, antimitochondrial autoantibodies, autoimmune hemolytic anemia, primary biliary cirrhosis, Sjögren syndrome

## Abstract

**Rationale:**

Primary biliary cholangitis (PBC) is a rare autoimmune cholestatic liver disease. It is often associated with extrahepatic autoimmune disorders. However, the concurrence of PBC and Sjögren syndrome (SS) with the subsequent onset of autoimmune hemolytic anemia (AIHA) is extremely rare.

**Patient concerns:**

This study investigated a 60-year-old woman admitted to our hospital with complaints of xerostomia for 5 years, pruritus for 3 years, and abnormal liver function for 3 months.

**Diagnoses:**

The patient was suffering from typical clinical PBC and SS, and developed decompensated liver cirrhosis after 32 months of ursodeoxycholic acid (UDCA) therapy. In May 2018, she was readmitted to the hospital with a high fever of 39 °C, coughing, and sever fatigue without remission after 3 days of cephalosporin antibiotic therapy. During the clinical course of PBC, her antimitochondrial antibodies (AMA) titers fluctuated from 1:1000 to negative and then to weakly positive, determined by indirect immunofluorescence (IIF), immunoblotting, and enzyme-linked immunosorbent assay (ELISA) based on recombinant mitochondrial antigens; furthermore, her titers of anti-gp210, an antinuclear antibody (ANA), increased sharply. Laboratory tests and imaging were performed to diagnose PBC and SS in September 2015. However, she was subsequently diagnosed with AIHA after 32 months of UDCA therapy based on the identification of pancytopenia, increased reticulocyte (RET) count, and a positive result from the direct Coombs test.

**Interventions:**

UDCA, hepatic protectant, albumin infusion, chest drainage, rational antibiotic use, diuretics, and methylprednisolone were used to treat the patient.

**Outcomes:**

Liver cirrhosis was complicated by the development of AIHA, which became severe at 42 months of follow-up.

**Lessons:**

This is the first case report showing a patient with comorbid PBC and SS, as well as the sequential development of AIHA with decreased AMA and increased anti-gp210 titers; this may have been due to immunodeficiency. These findings stress the importance of the serological screening of ANA profile, as well as repeated measurement of ANA and AMA to track PBC progression and prognosis.

## 1 Introduction

Primary biliary cholangitis (PBC, formerly called primary biliary cirrhosis) is a chronic autoimmune liver disease.^[1]^ It is characterized by elevated serum alkaline phosphatase, diagnostic autoantibodies targeting the mitochondria, and PBC-specific antinuclear autoantibodies (ANA), as well as the histopathological identification of liver granulomas around the bile ducts, resulting in cholestasis, portal inflammation, and fibrosis that may lead to cirrhosis and eventually to liver failure.^[2]^ Ursodeoxycholic acid (UDCA) is recommended as the first-line medicine for the treatment of PBC worldwide.

Antimitochondrial autoantibodies (AMA), detected by indirect immunofluorescence (IIF) in rodent kidney, liver, and stomach tissues, are the classical specific serological markers for PBC and are found in 90% to 95% of PBC patients.^[3]^ In addition to AMA, PBC-specific ANA, including anti-sp100 and anti-gp210, are present in over 30% of PBC patients found to be negative for AMA. The anti-gp210 antibody is a PBC-specific ANA that shows a membranous/rim-like pattern when examined by IIF; it targets a protein localized to the nuclear pore membrane and is associated with an increased risk of severe cholestasis or progression to hepatic failure.^[4,5]^

It has been demonstrated that the detection of AMA and PBC-specific ANA were correlated with disease severity, while the autoantibody titers did not vary significantly during follow-up; this suggests that AMA and PBC-specific ANA are stably autoantibodies that are not influenced by UDCA treatment.^[6]^ Furthermore, the sustained antibody response to gp210 was closely associated with the severity of interface hepatitis, in which anti-gp210 titers were sustained at high levels, and in which anti-gp210 status changed from positive to negative under UDCA therapy.^[4]^

PBC may be comorbid with many other autoimmune disorders, and Sjögren syndrome (SS) is one of the most frequently reported.^[7,8]^ Autoimmune hemolytic anemia (AIHA) is a fairly uncommon disorder characterized by the development of anti-erythrocyte autoantibodies and the destruction of erythrocytes; it is known to lead to moderate or severe anemia. The diagnosis of PBC concomitant with SS and AIHA is extremely rare. To our knowledge, only 5 cases have been reported until now.^[9]^ Herein, for the first time, we report a patient diagnosed with PBC and SS who developed AIHA after 32 months of UDCA therapy with decreased AMA titers and increased anti-nuclear rim antibody titers, specifically anti-gp210. The patient has provided informed consent for publication of the case.

## 2 Case presentation

In June 2015, a 60-year-old woman underwent a health examination; she was found to have high levels of alanine transaminase (ALT, 210 U/L) and aspartate transaminase (AST, 182 U/L). Two months later, she sought medical attention at a local hospital; the laboratory test data showed significantly increased levels of ALT (220 U/L), γ-glutamyltransferase (GGT, 618 U/L), and alkaline phosphatase (ALP, 779 U/L), and immunological testing revealed an ANA titer of 1:320. Hepatitis viral markers were all negative.

In September 2015, she visited our hospital for further treatment with complaints of xerosthomia for 5 years, pruritus for 3 years, and abnormal liver function for 3 months. The laboratory test data at admission showed elevated serum immunoglobulin M (IgM, 7.04 g/L). The AMA and ANA titers were determined by IIF (Liver Mosaic, EUROIMMUN Medizinische Labordiagnostika AG, Lübeck, Germany) to be 1:1000 respectively. The main patterns of ANA were anti-centromere antibodies (ACA); however, anti-nuclear rim antibody was weakly positive, with a low titer of 1:100. The specificity of anti-nuclear rim antibody was found to be anti-gp210, as determined by immunoblotting. The most suitable titration interval was obtained with a dilution factor of 3.162 (the square root of 10). In this way, every second dilution step resulted in a concentration with decreasing powers of 10 (1:10, 1:32, 1:100, 1:320, 1:1000, 1:3200, 1:10,000, etc.). EUROIMMUN recommends incubating samples at a dilution of 1:100, and a serum titer of 1:100 or greater is defined as a positive reaction. Further screening for ANA profiles and autoimmune liver diseases by immunoblotting revealed that the patient was strongly positive for anti-centromere protein B (anti-CENP B), moderately positive for anti-Ro52 and anti-nucleosome (anti-Nuk), positive for anti-SSA, and weakly positive for anti-gp210 and AMA-M2. AMA-M2 was 124.0 RU/mL (normal range: <25 RU/mL) according to the results of enzyme-linked immunosorbent assay (ELISA). The patient was diagnosed with PBC associated with SS and treated with UDCA (500 mg twice daily).

Twenty-five days into therapy, in October 2015, she was hospitalized at our institutions owing to nausea, abdominal distention, anorexia, and pruritus. On admission, physical examination identified severe jaundice, and liver palms, indicating chronic liver disease. We did not identify hematemesis, melena, spider nevi, abdominal pain, percussion pain in the liver area, hepatosplenomegaly, or edema, and the fluid wave test was negative. She had no hypertension, diabetes, history of drug abuse, history of alcohol abuse, hepatitis, or familial or occupational exposure history. She was allergic to seafood and was diagnosed with rhinitis 5 years prior, which was cured 4 years prior. Analysis of her seromarkers ruled out hepatitis A, B, C, and E; cytomegalovirus; and human immunodeficiency virus infections. Liver function results suggested cholestasis (TBil, 42.5 μmol/L; DBil, 18.6 μmol/L) and her levels of ALT, AST, GGT, and ALP were decreased compared with baseline levels but still more than double the upper normal limit (Table 1). Full blood differential counts indicated pancytopenia (red blood cells [RBC]: 3.03 × 10^12^/L, white blood cells [WBC]: 2.28 × 10^9^/L, hemoglobin: 98 g/L, platelets: 88 × 10^9^/L). Magnetic resonance cholangiopancreatography (MRCP) showed evidence of intrahepatic cholangitis, cirrhosis, splenomegaly, and mild ascites. Gastroscopy indicated mild esophageal varicose veins and portal hypertensive gastropathy.

**Table 1:**
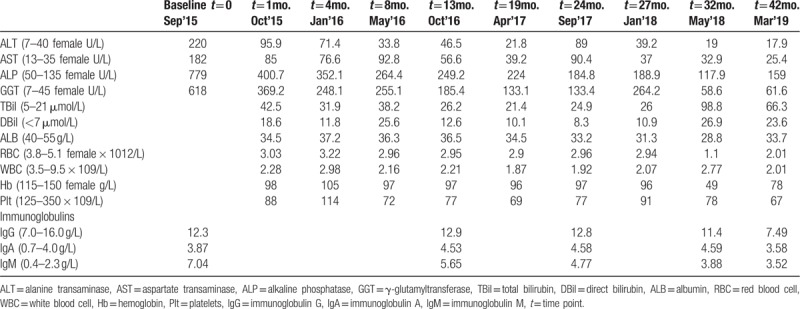
Routine laboratory results before and after UDCA therapy.

**Figure 1:**
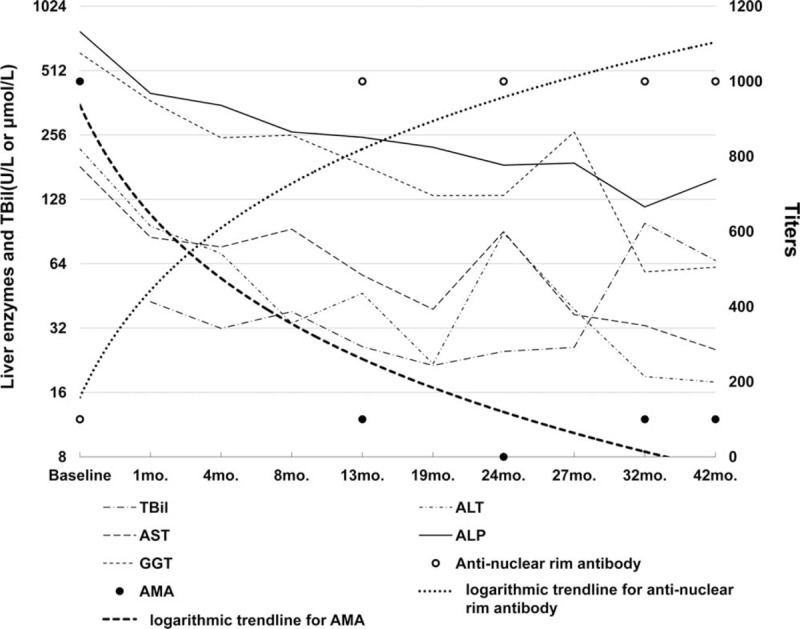
Clinical course of the patient. Trends showing changes in liver enzymes, bilirubin, and the titers of anti-nuclear rim antibodies and AMA. AMA = antimitochondrial antibodies.

During the following 32 months, the patient was treated with UDCA and hepatic protectant. Her condition showed limited improvements; she displayed persistent disease activity, as assessed by serum biochemistry, that indicated cholestatic hepatitis with impaired liver function (Table 1, Fig. 1). The patient was readmitted to our hospital in May 2018 owing to a high fever of 39 °C, coughing and severe fatigue without remission after 3 days of cephalosporin antibiotics therapy from a local hospital. On admission, jaundice, liver palms, a thick lung respiratory sound, splenomegaly, and suspicious fluid wave results were noted via physical examination. Chest CT images showed inflammation on the middle lobe of the right lung and the upper lobe of left lung, as well as right pleural effusions and thickening of the right pleura. An abdominal ultrasound showed evidence of cirrhosis, gallstones, splenomegaly, and mild ascites. Full blood differential counts still indicated pancytopenia (RBC: 1.10 × 10^12^/L, WBC: 2.77 × 10^9^/L, hemoglobin: 49 g/L, platelets: 78 × 10^9^/L). The reticulocyte (RET) count increased to 244.1 × 10^9^/L (normal range: 24–84 × 10^9^/L), and the direct Coombs test was positive. A bone marrow biopsy revealed that the erythroid hyperplasia was highly active. Repeated laboratory tests confirmed the presence of both ACA and anti-nuclear rim antibody by IIF, as well as the presence of anti-CENP-B, anti-SSA, anti-Ro52, and anti-gp210 by immunoblotting over the follow-up period of 32 months (Table 2). It is worth noting that AMA titers fluctuated from 1:1000 to negative and then to weakly positive, while the titers of anti-nuclear rim antibodies (specifically anti-gp210) increased sharply from weakly positive to strongly positive according to both IIF and immunoblotting (Table 2, Fig. 1). The fluctuations in the AMA and anti-gp210 titers were also confirmed using AMA-M2 ELISA, and using anti-M2–3E and anti-gp210 immunoblotting with different kits based on recombinant proteins. After combining the clinical findings with these results and consulting the hematology department of another hospital, the patient was finally diagnosed with AIHA in PBC associated with SS. By improving her liver function through albumin infusion, chest drainage, rational antibiotic use, diuretics, and methylprednisolone, the patient's clinical symptoms improved, and she was advised to seek further medical treatment for AIHA after discharge.

**Table 2:**
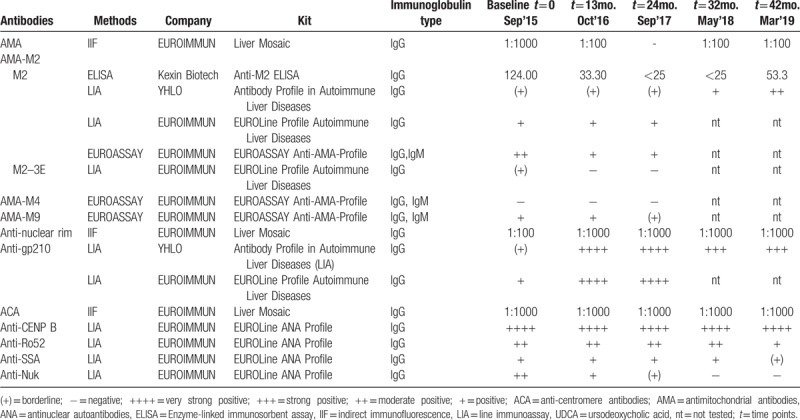
Comparative ANA and AMA profiles in different kits before and after UDCA therapy.

In March 2019, she was followed up in the outpatient department of our hospital. Physical examination and laboratory tests indicated that she still displayed hepatic decompensation, although the symptoms of cholestasis had been improved, as shown in Table 1 and Fig. 1. Anti-nuclear rim antibody and ACA were strongly positive with titers of 1:1000 respectively, while AMA was weakly positive with a low titer of 1:100. There was still evidence of severe AIHA, including pancytopenia, a positive result from the direct Coombs test, and an increased RET count (161 × 10^9^/L).

## 3 Discussion

Herein, we present a rare case of a patient diagnosed with PBC and SS, as well as comorbid AIHA during follow-up; the patient showed decreased AMA titers and increased anti-gp210 titers. PBC is a progressive disease with an insidious onset, resulting in the destruction of epithelial cells in the small intrahepatic bile ducts, leading to cholestasis and cirrhosis. A diagnosis of PBC should be suspected in patients with chronic cholestasis after excluding other major causes of liver disease, particularly in a middle-aged woman with an unexplained elevated serum ALP.^[10]^ AMA is highly disease-specific and an excellent diagnostic marker of PBC; it can be found in 95% of PBC patients, and often precedes the onset of liver damage by several years, even in individuals who are asymptomatic and do not have any other evidence of chronic liver disease.^[2]^ Although the mechanisms leading to the generation of AMA are unknown, it has been postulated that chemicals of environmental origin may trigger xenobiotic-induced and/or oxidative modification of mitochondrial autoantigens; this is a critical step leading to the breakdown of tolerance to the lipoyl domain of the E2 subunit of pyruvate dehydrogenase (PDC-E2) and are present before the onset of clinical disease.^[11]^ The presence or absence of AMA, rather than the magnitude of antibody level, is most important for diagnosis, and AMA titers do not appear to be correlated with disease severity or prognosis in patients with PBC.^[10,12]^ However, the fluctuations in the AMA titers in this case were intriguing. At baseline level, the AMA titer was 1:1000 by IIF; this then sharply decreased to weakly positive of 1:100 after 12 months of UDCA therapy. Moreover, AMA was undetectable by IIF after 2 years of UDCA treatment, and then reappeared at a low titer of 1:100 after UDCA treatment for 32 months; this was sustained until 42 months of follow up. Repeated tests by immunoblotting and ELISA confirmed the changes in the AMA titers with target antigen of AMA-M2 (PDC-E2 and 2-oxoglutaric acid dehydrogenase complex) and a hybrid containing the 3 E2-subunits (M2-3E, also known as BPO or MIT3; a fusion protein of the immunogenic lipoyl domains of branched-chain oxoacid dehydrogenase, pyruvate dehydrogenase, and oxoglutarate dehydrogenase) which possess high levels of diagnostic sensitivity.^[13–15]^ We identified weak correlations between the values obtained using these methods; furthermore, the results were often inconclusive regarding whether AMA was positive or negative, especially after 24 months of UDCA treatment (Table 1). Even when the same manufacturer's kits were used, the AMA-M2 results were not very consistent. Although there is no evidence to suggest that a concentration of AMA above the diagnostic threshold holds any prognostic significance, and although repeated measurement is not recommended once a clear-cut diagnosis is established,^[16]^ in this case, the titers of AMA and AMA-M2 decreased with disease progression during UDCA therapy and were undetectable by both IIF and ELISA at some points. Furthermore, the anti-AMA-Profile showed that anti-M4 remained negative during the clinical course, while anti-M9 was detectable and changed from positive to weakly positive over the follow-up period. Anti-M9 can occur in the absence of anti-M2, and may be helpful for the diagnosis of early and asymptomatic PBC; in contrast, anti-M4 is always associated with anti-M2.^[17,18]^

PBC sera may contain a number of autoantibodies targeting specific constituents of the nuclear envelope. There is evidence to suggest that PBC-linked ANA (in particular, anti-gp210/anti-nuclear rim antibody) may be associated with more rapidly progressive disease and disease which is less responsive to UDCA therapy.^[5,19]^ Early studies showed that the appearance and titer of anti-gp210 in the patients with PBC did not vary from the time of diagnosis and throughout their clinical course.^[20]^ However, anti-gp210 antibody changed to negative in 20% of anti-gp210 positive patients after UDCA treatment; therefore, the monitoring of anti-gp210 antibodies titers in patients treated with UDCA could be significant, as suggested by Nakamura et al,^[4,21]^ who determined that the progression to hepatocellular failure was lower in patients undergoing UDCA treatment with a decreased titer of anti-gp210 antibodies; furthermore, they identified the increased expression of the gp210 antigen in the epithelial cells of patients with PBC, and that its expression level was positively correlated with the histological activity of the disease, suggesting a pathogenic role of the gp210 antigen itself. In the current case, the titer of anti-gp210 was weakly positive at baseline, then increased to strongly positive at the end of 12 months into UDCA therapy; the level was sustained during follow-up, as confirmed by both IIF and immunoblotting. The patient had developed decompensated liver cirrhosis after 32 months of UDCA treatment. More interestingly, the increased titers of anti-gp210 were accompanied by a decreased AMA titer, and these changes showed a cross trend. The current case indicated that the serum titers of AMA and anti-gp210 might be correlated with disease severity or prognosis, and that repeated measurements are important even after a clear-cut diagnosis had been established.

For the IIF method has the lowest sensitivity, approximately 5% to 10% of patients with clinical, biochemical, and histological features compatible with PBC do not have detectable AMA. AMA-negative PBC was not “true” negative in which AMA could not be detected by currently available techniques.^[22]^ In some other AMA-negative PBC patients, antibodies against the major M2 components were identified using ELISA or Western blotting, and over 15% of AMA-negative sera based on IIF showed reactivity to MIT3.^[23]^ Compared with AMA-positive PBC patients, AMA-negative PBC patients tend to have more non-hepatic autoimmune conditions and worse health-related quality of life in both social and emotional domains.^[24,25]^

Nearly all AMA-negative PBC patients have PBC-specific antinuclear antibodies, including sp100 and gp210, which are present in over 30% of PBC patients negative for AMA by IIF.^[10]^ The generation of ANA may be due to the molecular mimicry of mitochondrial peptides. One hypothesis is that molecular mimicry can be operative in the diversification of autoantibody-repertoire from PDC-E2 to gp210 during the course of disease progression in PBC, because an immunodominant T-cell autoepitope from human PDC-E2 can cross-react to mimicry peptides derived from nuclear antigens such as human gp210.^[26–29]^ According to this hypothesis, the breakdown of immunological tolerance to mitochondrial antigens such as PDC-E2 is the first critical step at a very early stage of the disease, and is not enough for the progression to hepatic failure (hepatic failure-type progression). The additional breakdown of immunological tolerance to nuclear antigens such as gp210 at an early stage (stage 1–2) is the second critical step for this type of progression.^[21]^ Based on the above, the current case verified the hypothesis that AMA-positive PBC could be transformed to AMA-negative PBC for a limited period during disease progression because the AMA status changed from an initial positive to negative or weakly positive after the initiation of UDCA therapy. We speculated that AMA-negative PBC with transient detectable AMA at a very early stage of the disease that could not be detected for a while or during the remainder of the clinical course; furthermore, PBC-specific antinuclear antibodies such as anti-gp210 appeared in place of AMA; we believe that this case showed a very rare manifestation of the disease.

PBC is principally associated with other autoimmune conditions reflecting shared immunogenetic susceptibility.^[30,31]^ The strongest association is with SS (most frequently secondary “sicca complex,” although primary SS[pSS] is also associated),^[16]^ occurring in proportions ranging from 3.5% to nearly 100% of cases.^[32]^ In this case, the patient complained of a dry mouth for 5 years prior to admission, implying that she may have suffered from SS before PBC. PBC and SS share a common immunopathogenesis in which genetics and environmental factors interact resulting in the onset of salivary or biliary epithelial cell apoptosis, contributing to the breakdown of tolerance to self-antigens exposed to the apoptotic blebs.^[33]^

AIHA is a rare autoimmune disorder with an incidence of 1 to 3 cases per 100,000 people per year, and secondary AIHA accounts for 20% to 80% of cases, according to diverse report series^[34]^; in AIHA, autoantibodies directed against erythrocytes antigens lead to their increased destruction.^[35]^ A previous case report suggested that the occurrence of AIHA in pSS might be associated with the exacerbation of autoimmune cholangiopathy.^[36]^ A recent large-scale retrospective screening study of 565 pSS patients indicated that 5 out of the 16 patients with both SS and AIHA also suffered from PBC. The coexistence of pSS and PBC was observed in 31.3% of the SS-AIHA patients, markedly more frequent than that in the SS only group (4.7%). PBC was more prevalent in patients with SS-AIHA than in SS patients without AIHA (*P* = .007). Among pSS patients, the existence of PBC, cytopenia, or hypocomplementemia suggested a higher risk of suffering from AIHA.^[9]^

It is well known that people with 1 autoimmune disease are more prone to developing other autoimmune diseases.^[37]^ To date, there is no clear pathogenic mechanism for the extrahepatic manifestations of PBC, which are generally autoimmune in nature. The pathogenesis of these conditions includes a common mechanism involving both innate and adaptive immune responses targeting cholangiocytes and different extrahepatic tissues.^[32]^ Moreover, in patients with PBC, any associated extrahepatic manifestations were unrelated to the onset of major events during the follow-up (complications of end-stage liver disease, hepatocellular carcinoma, or extrahepatic cancer), and they did not affect patient survival.^[8]^ For PBC patients who are more likely to develop extrahepatic manifestations, awareness of their increased risk and close screening are imperative.^[37]^ The ANA profile can be used to screen for common autoimmune diseases. In this case, the patient had an autoimmune disorder showing detectable ACA and anti-Ro52, while anti-Nuk gradually decreased and then disappeared during the course of PBC.

ACA are found in approximately 50% of patients with CREST syndrome (calcinosis, Raynaud phenomenon, esophageal dysmotility, sclerodactyly, and telangiectasia), a benign variant of systemic sclerosis.^[38]^ They are found not only in approximately 20% to 30% of PBC patients, but also in patients with other autoimmune diseases including systemic sclerosis, SS, systemic lupus erythematosus, rheumatoid arthritis, and Raynaud phenomenon without definite rheumatic disorders in lower frequencies.^[39–41]^ ACA-positive PBC patients can be affected by limited systemic sclerosis and Raynaud phenomenon^[42]^; they also seem to progress more rapidly to liver failure, have higher serum alkaline phosphatase levels, and show a greater frequency of portal hypertension with the development of esophageal varices or hepatocellular carcinoma.^[43,44]^ The status of anti-gp210 and ACA may predict not only the long-term prognosis, but also the biochemical response to treatment in PBC patients.^[5,21]^ Given the propensity for patients with PBC to develop extrahepatic manifestations, and because some of these extrahepatic manifestations can lead to diseases with a poor prognosis, vigilant screening and close follow-up will lead to the prompt identification and treatment of these diseases.^[37]^

In summary, we present an extremely rare Chinese case of PBC accompanied by SS and sequential development of AIHA after 32 months of UDCA therapy. Unlike previously reported cases, the patient's clinical course was complicated by decreased titers of AMA and increased titers of anti-gp210, verifying the hypothesis that AMA-positive PBC can transform to AMA-negative PBC for some time. The purpose of presenting this case was to provide insights into the concurrence of PBC, SS, and AIHA, as well as to stress the importance of serologic screening of ANA profile and repeated measurements of ANA and AMA for PBC progression and its prognosis. Prompt recognition of extrahepatic diseases should be raised which can lead to improved patient outcomes and quality of life.

## Acknowledgments

The authors would like to thank EUROIMMUN (China) for the great support in the repeated laboratory tests for autoantibodies by kits of “EUROLine Profile Autoimmune Liver Diseases” and “EUROASSAY Anti-AMA-Profile.”

## Author contributions

**Conceptualization:** Dan-Tong Zhao, Hui-Ping Yan.

**Data curation:** Dan-Tong Zhao, Yan-Min Liu, Ying Han, Hai-Ping Zhang, Yan Zhao, Hui-Ping Yan.

**Formal analysis:** Dan-Tong Zhao, Yan-Min Liu, Ying Han, Hai-Ping Zhang, Yan Zhao, Hui-Ping Yan.

**Funding acquisition:** Dan-Tong Zhao, Yan Zhao, Hui-Ping Yan.

**Investigation:** Dan-Tong Zhao, Yan-Min Liu, Ying Han, Hai-Ping Zhang, Hui-Ping Yan.

**Methodology:** Dan-Tong Zhao, Yan-Min Liu, Ying Han, Hai-Ping Zhang, Hui-Ping Yan.

**Project administration:** Dan-Tong Zhao, Yan-Min Liu, Hui-Ping Yan.

**Resources:** Dan-Tong Zhao, Yan-Min Liu, Ying Han, Hai-Ping Zhang, Hui-Ping Yan.

**Supervision:** Yan-Min Liu, Yan Zhao, Hui-Ping Yan.

**Validation:** Hui-Ping Yan.

**Visualization:** Dan-Tong Zhao.

**Writing – original draft:** Dan-Tong Zhao.

**Writing – review & editing:** Hui-Ping Yan.
